# Frequently Touched Sites in the Intensive Care Unit Environment Returning 100 Colony-Forming Units per Surface Area Sampled Are Associated With Increased Risk of Major Bacterial Pathogen Detection

**DOI:** 10.7759/cureus.68317

**Published:** 2024-08-31

**Authors:** Matthew D Koff, Franklin Dexter, Soyun M Hwang, Brendan T Wanta, Jonathan E Charnin, Randy W Loftus

**Affiliations:** 1 Anesthesiology, Dartmouth Hitchcock Medical Center, Lebanon, USA; 2 Anesthesia, University of Iowa, Iowa City, USA; 3 Anesthesiology and Perioperative Medicine, Mayo Clinic, Rochester, USA

**Keywords:** bacterial pathogens, colony-forming units, intensive care unit, frequently touched sites, 100 cfu, threshold, icu, cleaning, quality

## Abstract

Background: A threshold for surface hygiene has not been defined for the healthcare arena. We aimed to identify the magnitude of bacterial contamination of frequently touched sites in the intensive care unit (ICU) environment that could be used to guide quality improvement initiatives.

Methods: Nineteen patients in a mixed ICU environment (providing care for medical and surgical patients) were followed from admission for 72 hours in 2010. Baseline cultures of frequently touched environmental sites were obtained at time zero following active decontamination and at 12, 24, 48, and 72 hours without further disinfection. We tested for an association of environmental reservoirs returning ≥ 100 colony-forming units (CFU) per surface area sampled with major bacterial pathogen detection.

Results: There were 446 ICU room, day, and reservoir combinations sampled from 19 patients. There were pathogens detected in 40% (79/199) of samples with ≥ 100 CFU vs. 14% (35/247) of samples returning < 100 CFU. The relative risk was 2.80 (95% CI: 1.97-3.98, P <0.0001). The odds ratio adjusted for time in hours was 3.11 (95% CI: 1.84-5.34, P < 0.0001).

Conclusions: Frequently touched ICU environmental sites returning ≥ 100 CFU are associated with major bacterial pathogen detection. This threshold for surface hygiene can be used to ensure compliance with ICU environmental cleaning protocols and to guide quality improvement initiatives.

## Introduction

Despite the use of sterilization/disinfection protocols, advances in surgical techniques, and the development of evidence-based guidelines, healthcare-associated infections (HAIs) remain a major risk to hospitalized patients [1-3]. Although multifaceted, HAIs are frequently attributed to bacterial cross-contamination, often a consequence of poor compliance with infection control guidelines [4-8].

Within the hospital, there are multiple reservoirs that contribute to bacterial cross-contamination [9-14]. In the operating room (OR), the anesthesia machine has been associated with most high-risk bacterial transmission events involving patient intravenous stopcock sets (stopcocks) [15], and stopcock contamination events have been repeatedly associated with increased patient mortality [6,15]. Due to the known contribution of surface contamination to HAIs, the Centers for Disease Control and Prevention recommends that hospitals ensure compliance with environmental cleaning and disinfection protocols [16]. There are standards for acceptable levels of surface contamination for the food industry [17]. For example, swab samples that return > 100 colony-forming units (CFU)/10 cm^2^ indicate an unacceptable level of kitchen surface contamination in need of improvement [17]. Currently, there are no standards for surface bacterial contamination that can be used to guide quality improvement strategies in the healthcare arena.

Prior work has investigated a threshold for surface contamination in the OR. Swab samples of anesthesia workspace reservoirs returning ≥ 100 CFU per surface area sampled are associated with an increased risk of major bacterial pathogen isolation [18] and stopcock contamination [6] events versus reservoirs returning < 100 CFU. In one study, a reduction in surface contamination of the anesthesia machine below 100 CFU achieved via improved anesthesia provider hand hygiene was associated with a reduced incidence of stopcock contamination and HAIs [7]. In a cluster randomized study, anesthesia machine swab samples returning ≥ 100 CFU were used to provide feedback to improve the cleaning of the anesthesia machine [19]. When combined with improved provider hand hygiene, patient decolonization, and vascular care (e.g., intravenous injection port disinfection), there were substantial reductions in *Staphylococcus aureus* transmission and surgical site infections (SSIs) [19]. Those results were confirmed in a large postimplementation analysis involving over 800 surgical patients [20]. Thus, a solid body of evidence suggests that like the food industry [17], the 100 CFU threshold for surface contamination can be used to guide quality improvement in the OR [6,18-20].

However, it is unknown whether the 100 CFU threshold is relevant for other high-risk clinical environments. This threshold may be helpful for improved quality of surface disinfection in the intensive care unit (ICU) where major bacterial pathogens such as vancomycin-resistant *Enterococcus* (VRE) [21] and methicillin-resistant *Staphylococcus aureus* (MRSA) [22] are frequently transmitted from environmental surfaces to healthcare worker hands and patients [21,22]. In this study, we aimed to assess whether the 100 CFU threshold is relevant for high-touch environmental sites in the ICU. We hypothesized that the association of the 100 CFU threshold with major bacterial pathogen detection [18] would remain for the ICU even despite assessment during a different clinical context (2010 vs. 2017-2022) [18] potentially associated with a secular change in infection control practices.

## Materials and methods

This is a new analysis of historical data previously obtained during a prospective observational study conducted in a multidisciplinary ICU at Dartmouth Hitchcock Medical Center (DHMC) over 72 consecutive hours in 2010. The intent of this study was to further validate recent work in the OR that showed an association of a 100 CFU threshold for surface hygiene with major bacterial detection [18]. Approval was obtained from the institutional review board with no requirement for patient or provider consent (Committee for the Protection of Human Subjects (CPHS), Dartmouth College, Hanover, New Hampshire, CPHS #20655). The primary aim of the original study was to characterize bacterial transmission among ICU reservoirs.

Study design, sample collection, processing, and archiving were completed by Drs. Koff and Loftus (DHMC). A formal analysis was conducted by Dr. Dexter (University of Iowa), where data sharing involved email communication to Dr. Dexter from Drs. Koff/Loftus. The original draft was completed by Dr. Loftus at the Mayo Clinic in Rochester, MN, and shared via email communication with Drs. Hwang, Wanta, and Charnin at the Mayo Clinic in Rochester, MN, with Dr. Koff at DHMC and Dr. Dexter at the University of Iowa for their expert review and editing prior to submission. All authors reviewed and approved the final manuscript.

DHMC is a tertiary care and level 1 trauma center with a multidisciplinary medical/surgical ICU consisting of 26 patient beds. Beds 1-18 were located in a different ICU patient care bay than beds 19-26, and all rooms were single occupancy. Two dedicated critical care teams cared for all patients admitted into the ICU, and the critical care nursing-to-patient ratio was either 1:1 or 1:2. Physicians, nurses, and technicians underwent training regarding the five moments for hand hygiene (before touching a patient, before a procedure, after a procedure or body fluid exposure risk, after touching a patient, and after touching a patient's surroundings), as recommended by the World Health Organization (WHO) [5], and a hospital-wide infection control department actively monitored hand hygiene compliance. Nurses trained in aseptic practice were instructed to utilize claves or dead-end caps on all intravenous lumens, and barrier precautions were routinely utilized on patients with either suspected or known VRE or *Clostridium difficile* colonization or infection, but not for patients with other multidrug-resistant bacteria such as MRSA. All healthcare providers had access to wall-mounted, alcohol-based dispensers located on the wall immediately outside of each ICU patient room and to a bottle of 70% alcohol-based hand cleaner present on the medication cart. Observed provider hand hygiene compliance was recorded at approximately 80% during the study period. The ICU cleaning strategies routinely employed during the study period included terminal cleaning, thorough cleaning of the environmental surfaces with a quaternary ammonium compound (Dimension III™, Butcher’s, Sturtevant, WI) following patient discharge, and twice daily cleaning of the ICU medication carts.

All ICU rooms with patients undergoing mechanical ventilation were included in the study. The patient bed rail, infusion pump, monitor, ventilator knobs, and medication cart were considered frequently touched environmental sites [23]. Using aseptic technique (hat, mask, and donning gloves following hand hygiene), each site was sampled by the same technician using sterile polyester fiber-tipped applicator swabs moistened with sterile transport medium (ESwab, Copan Diagnostic Inc., Corona, CA) to roll several times over the selected areas followed by culturing on 5% sheep blood agar (SBA) plates (Thermo Fisher Scientific, Waltham, MA) with a zigzag pattern and swab rotation to detect both Gram-positive and Gram-negative bacteria [6,15]. The two sampling methods utilized in nonclinical environments include contact plates for smooth surfaces and swabbing for irregular surfaces [17]. We utilized swabs due to the irregularity of the surfaces [17] and sampled up to 10cm^2^. This technique of proven clinical utility [19,20] is more likely to remove the 10-20% of bacteria that would result in object contamination (e.g., provider's hands) following contact versus contact plates [17]. These sites were monitored in each ICU room immediately following active decontamination with a quaternary ammonium compound (Dimension III™, Butcher’s, Sturtevant, WI) at time zero and until 72 hours or discharge.

All blood agar plates were inoculated at the time of sample collection, taken to the lab immediately following with care to ensure that the plates remained covered, and they were incubated under aerobic conditions at 36.5°C for 48 hours. Microorganisms were quantified according to standard laboratory methods [17,24]. Bacterial isolates were identified by colony morphology, Gram stain (Thermo Fisher Scientific, Waltham, MA), simple rapid tests (e.g., coagulase, catalase, and oxidase (Thermo Fisher Scientific)), the Dade Behring MicroScan (San Diego, CA), and conventional and chromogenic tests utilizing pH changes, substrate utilization, and growth in the presence of antimicrobial agents (e.g., methicillin and vancomycin) after 24 hours of incubation at 36.5°C (Thermo Fisher Scientific) [24].

Statistical analysis

High-touch environmental CFUs were stratified as ≥ 100 CFU or < 100 CFU. Too numerous to count (≥500) were treated as ≥ 100 CFU. The proportion of cultures with major bacterial detection was compared for samples returning ≥ 100 vs. < 100 CFU via exact logistic regression (Stata v18.5, StataCorp, College Station, TX). The exact logistic regression was repeated controlling for time from decontamination, treated linearly as done by the Cochran-Armitage test. P < 0.05 was considered statistically significant.

## Results

There were 446 ICU room, day, and reservoir combinations sampled from 19 patients. Patient demographics are shown in Table 1.

**Table 1:** Patient demographics. MSSA: methicillin-sensitive *Staphylococcal aureus*; MRSA: methicillin-resistant *Staphylococcal aureus*; GI: gastrointestinal; ICH: intracranial hemorrhage; Ab = abdominal; ICU: intensive care unit; spp. = species.

ICU room	Sex	Admitting diagnosis	Days patient was in the ICU prior to the culture of the room	Existing patient cultures
1	Female	Septic shock	5	MSSA
3	Male	GI bleed	4	MSSA
4	Male	Respiratory failure	7	None
5	Female	Pneumonia	12	*S. epidermidis*, *Candida spp*.
6	Female	Esophagectomy	14	*Enterococcus spp*.
7	Male	GI bleed	19	*Serratia spp*., MSSA, *E. coli*
8	Female	Respiratory failure	7	None
10	Male	Vascular bypass	5	Coagulase-negative staphylococcus
11	Female	Small bowel obstruction	5	*Klebsiella spp*.
12	Male	Pancreatitis	16	*Candida spp*., *Enterobacter spp*.
13	Male	Drug overdose	4	None
17	Male	Necrotizing fasciitis	18	MSSA, *Pseudomonas spp*.
18	Female	Trauma	1	None
19	Male	Ab sepsis	4	*Pseudomonas spp*., coagulase-negative staphylococcus
21	Male	Trauma	3	None
23	Female	ICH	10	MRSA
24	Male	Open ankle fracture	10	None
25	Male	Gangrene	150	MRSA, *Proteus spp*., coagulase-negative staphylococcus, *E. coli*, *Serratia spp*.
26	Female	Sepsis	7	MSSA, coagulase-negative staphylococcus

**Figure 1:**
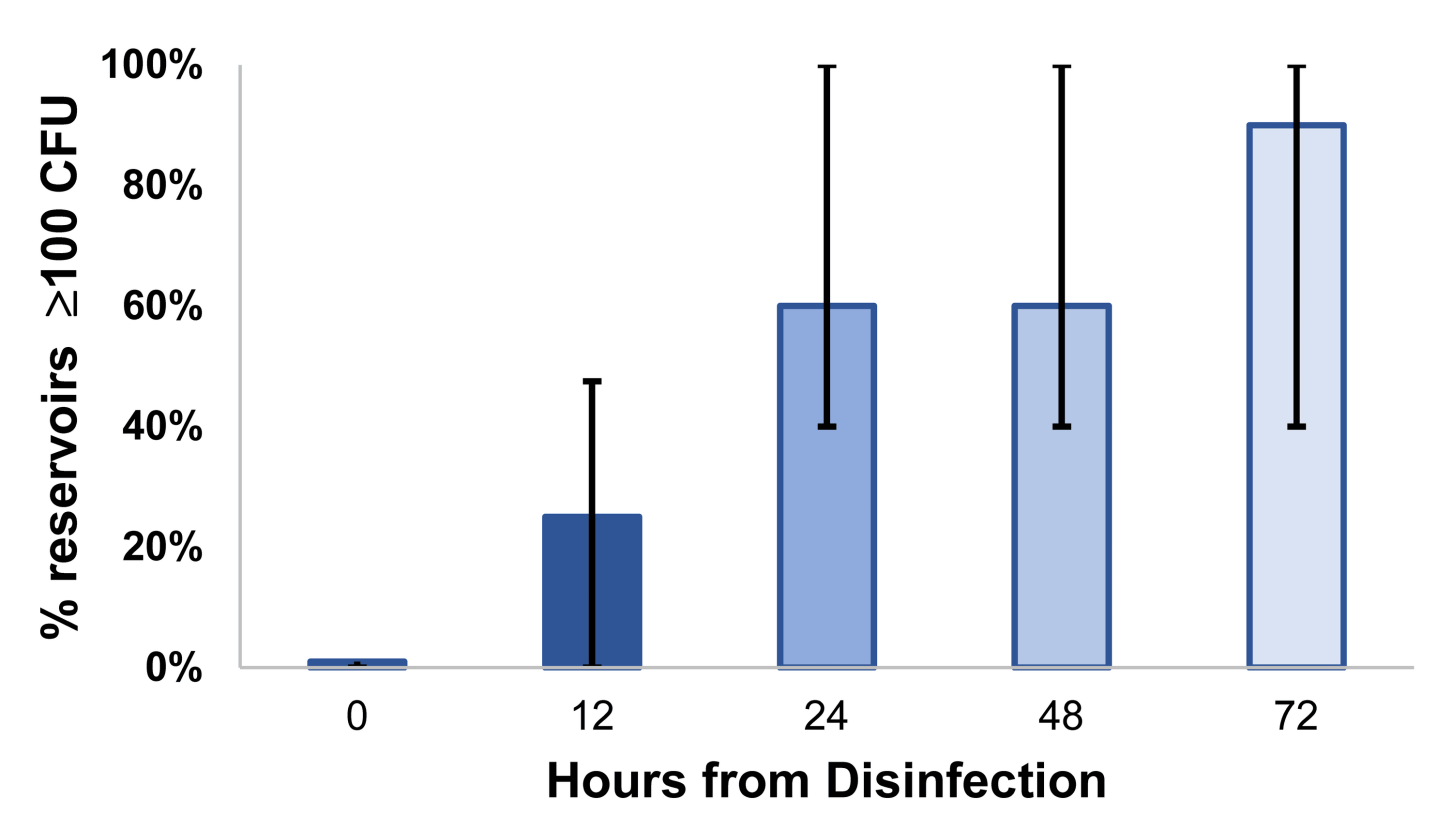
The proportion of frequently touched intensive care unit reservoirs reaching 100 colony-forming units per surface area sampled over the 72-hour period following active decontamination The proportion of reservoirs that reached or exceeded 100 colony-forming units (CFU) over time (0-72 hours). The median is plotted, along with 25th and 75th percentiles. The sample sizes are 20 patients (in 19 rooms) for 0, 12, and 24 hours, and 19 for 48 and 72 hours (one patient was discharged prior to the 48- and 72-hour cultures). Each 12-hour increase was associated with an increase in percentage incidence of ≥ 100 CFU, Jonckheere-Terpstra test for trend P < 0.0001. By 24 hours, many reservoirs (median 60%, IQR 40% to 100%) had reached ≥ 100 CFU, suitable for the current study. The low incidence of contamination at 12 hours supports once-daily cleaning in the ICU, which matches common practice and suggests the generalizability of the data.

There were pathogens detected in 40% (79/199) of samples with ≥ 100 CFU vs. 14% (35/247) of samples returning < 100 CFU. The relative risk for major bacterial detection was 2.80 (95% CI: 1.97-3.98, P < 0.0001). The odds ratio for major bacterial detection was 3.98 (95% CI: 2.47-6.49, P < 0.0001). Samples with progressively greater contamination were from more hours since decontamination (Figure 1).

Using exact logistic regression to control for time, the adjusted odds ratio was 3.11 (95% CI: 1.84-5.34, P < 0.0001).

## Discussion

The CDC recommends hospital adherence to environmental cleaning protocols to reduce the risk of bacterial cross-contamination [16]. Environmental samples returning > 100 CFU/10 cm^2^ via a swabbing technique represent an unacceptably high level of contamination for nonclinical environments such as kitchen surfaces [17]. Currently, there are no standards for surface hygiene that can be used to guide quality improvement initiatives in the clinical arena. In this study, we aimed to further validate a 100 CFU threshold of surface hygiene previously shown to be associated with major bacterial detection in the OR environment [18].

Prior work has confirmed an association of 100 CFU/surface area sampled with stopcock contamination events that are repeatedly associated with increased patient mortality [6,15]. In addition, this threshold was associated with an increased risk of major bacterial pathogen detection in a more recent study involving nine geographically dispersed hospitals in the United States and over 30,000 anesthesia workspace reservoirs [18]. This study adds to the current body of literature by providing an assessment of the association in a different clinical context to further validate the association.

We examined the association of the 100 CFU threshold with major bacterial detection in an ICU environment in 2010. We found that the association of the 100 CFU threshold with major bacterial pathogen detection remained true for the frequently touched environmental reservoirs observed. Thus, these results do not support an alternative explanation for the earlier association among anesthesia reservoirs [18] that the results were limited to the OR setting, to the study time period, and/or to specific infection control policies and/or procedures. Importantly, these study results in combination with prior OR data [18] and the use of the 100 CFU threshold to assess the quality of cleaning in the food industry [17] support the use of the 100 CFU threshold to broadly ensure compliance with hospital cleaning protocols [16]. We also examined the change in the magnitude of reservoir contamination over the 72-hour period following active decontamination with a quaternary ammonium compound. While most reservoirs reached the threshold by 24 hours, supporting once-daily cleaning, approximately 20% reached the threshold by 12 hours. Thus, an increased frequency of cleaning to twice daily may be indicated. For example, increased frequency of cleaning could be used to address infectious disease outbreaks or clusters of infections.

There are practical applications for use of the 100 CFU threshold that can be immediately employed. In one study, anesthesia workspace reservoirs meeting or exceeding the 100 CFU threshold were mapped to assess the fidelity of a variety of infection control measures for the anesthesia workspace [25]. Clark et al. increased the frequency of cleaning via the incorporation of a postinduction wipe-down strategy of anesthesia equipment. These efforts reduced the proportion of measured reservoirs returning ≥ 100 CFU, 46% usual care vs. 12% with quality improvement [26]. This approach could be used for improved frequency of cleaning for frequently touched sites in the ICU environment (e.g., ventilator knob). Triangular ultraviolet-C (UV-C) irradiation treatment for 21 minutes can attenuate the more pathogenic *S. aureus* sequence type 5 (ST5) strain characteristic that is associated with increased risk of transmission of antibiotic resistance between operating rooms on different dates (longitudinal transmission) [27]. This approach found to be non-inferior to surface disinfection in the laboratory setting [27] could be employed to achieve improved quality of cleaning via augmentation of surface disinfection cleaning efforts. Thus, the 100 CFU threshold could be used to guide the implementation of these evidence-based improvement strategies to improve the quality of cleaning for hospital environments, including but not limited to the OR and the ICU. Broad integration of this threshold to achieve improved surface hygiene across a variety of hospital environments could be executed by application of the reported methodology to reservoir mapping to basic preventive measures. Reservoirs meeting or exceeding the threshold could then be reported via a simplistic, evidence-based dashboard [19,20,25] to stakeholders to provide impetus for the optimization of the respective measure. Such monitoring is proven to be cost-saving [28], practical [29], and applicable to a variety of pathogens [30].

Potential limitations

The intent of this study was to use archival data to further validate more recent findings [18]. The intact association despite the potential for differences in infection control practices in a different time period and in a different environment further validates the association [18]. As we examined a mixed ICU setting, further work could include assessment in different ICU settings (e.g., cardiovascular, trauma, and/or surgical neuro-intensive care units). Such work would also allow further assessment of the potential impact of secular change in infection control practices. The environmental sampling technique is proven; when utilized to provide feedback, it can reduce the proportion of environmental reservoirs returning 100 or more CFU, reducing infections as part of a multifaceted program [6,17,19,20). While contact plating may be a better approach for smooth surfaces, this would not have been practical for the irregular objects measured in this study. Further, the approach utilized would be more likely to recover the proportion of bacteria that are more likely to result in object contamination (e.g., provider hands) on contact, and in turn, cross-contamination [17]. Thus, the approach utilized for sampling strengthens the clinical relevance of the findings and supports the established threshold in the nonclinical arena [17]. We recognize that like all other sampling techniques, the swab technique utilized is not 100% efficient in collecting bacterial pathogens present on the studied environmental surfaces [17]. While we could have evaluated a different CFU magnitude, we chose 100 CFU given that it is already a standard in nonclinical arenas [17] and has proven clinical utility [18-20] in the OR. The study gap was a confirmation of relevance in a different clinical arena and time period to further validate the threshold prior to more widespread use. This was accomplished in this study. While it may seem logical that more CFU would equate to more major pathogens, as sampling methodology captures only some of the available pathogens (approximately 10-20%) on any given surface [17], the efficacy of a monitoring approach must be validated, not assumed. When considering the body of evidence that we have generated, for the OR [6,7,15,19,20,25-27] and now the ICU, in the context of established methodology for nonclinical arenas [17], we present in this manuscript an evidence-based approach, including target reservoirs, sampling techniques, and clinical outcomes, that can be employed for a novel monitoring system to improve environmental cleaning quality in the healthcare arena.

## Conclusions

In conclusion, frequently touched sites in the ICU environment that return ≥ 100 CFU are associated with major bacterial pathogen detection. This cleaning threshold should be used to guide improved quality and frequency of ICU environmental cleaning.
